# Supplementary material for: MamF-like proteins are distant Tic20 homologs involved in organelle assembly in bacteria
Source: Nat Commun. 2024 Dec 9;15:10657. doi: 10.1038/s41467-024-55121-0 (PMC11628618; doi:10.1038/s41467-024-55121-0)
Supplement: Supplementary file 1 — Supplementary Information [file 41467_2024_55121_MOESM1_ESM.pdf]

Supplementary Information

MamF-like proteins are distant Tic20 homologs involved in organelle assembly in bacteria

Anja Paulus[1], Frederik Ahrens[1], Annika Schraut[1], Hannah Hofmann[1], Tim Schiller[1], Thomas Sura[3], Dörte Becher[3], René Uebe[1,*]

Affiliations:

[1]Department of Microbiology, University of Bayreuth; Bayreuth, Germany.

[2]Microbial Proteomics, Institute of Microbiology, University of Greifswald; Greifswald, Germany.

*Corresponding author. Email: rene.uebe@uni-bayreuth.de

This PDF file includes:

Supplementary Note 1
Supplementary Figures 1-5
Supplementary Tables 1-4

Other Supplementary Materials for this manuscript include the following:

Supplementary Data 1-5
Source data file

Supplementary Note 1

Altered composition of the MM in MFP mutants

In addition to the eleven proteins that showed a significantly decreased abundance in MM fractions of strain ΔF3, our quantitative proteomic analyses also revealed that 26 proteins are significantly enriched compared to the WT (**Supplementary Data Table 4**). Among these proteins, the three MAPs MamC, MamW, and MamX showed a three-fold increase (**Fig. 3d**). While increased MamC levels could be confirmed by Western immunoblots (**Supplementary Fig. 5a**), the deletion of *mamC* or *mamW* had only very weak effects on magnetosome formation [1,2]. Since MamX, on the other hand, plays a role in magnetosome redox control [3], the enrichment of these MAPs does not seem to play a role for the ΔF3 phenotype. Beside these MAPs, 23 significantly enriched proteins have no known function in magnetosome biomineralization and are encoded outside the genomic magnetosome island that contains almost all MAP genes [2]. Strikingly, 13 of these proteins contain predicted secretory N-terminal signal sequences (SignalP 5.0) (**Supplementary Data Table 4**) [4] and thus likely reside within the periplasm or the outer membrane. To test for a putative role in magnetosome biogenesis, we fluorescently labeled three of the most strongly enriched proteins (**Supplementary Fig. 5b, c**). When produced in the WT, all proteins showed a peripheral cell localization, whereas mCherry-labeled MmeA, a MAP with a known N-terminal signal peptide [5], showed a magnetosome chain-like localization pattern. Thus, at least the tested proteins (MSR-1_01270, MSR-1_09970, MSR-1_21580) do not localize to the MM *in vivo*. We thus concluded that only proteins with a decreased abundance in ΔF3 MM extracts are relevant for our analyses.

Supplementary Figures

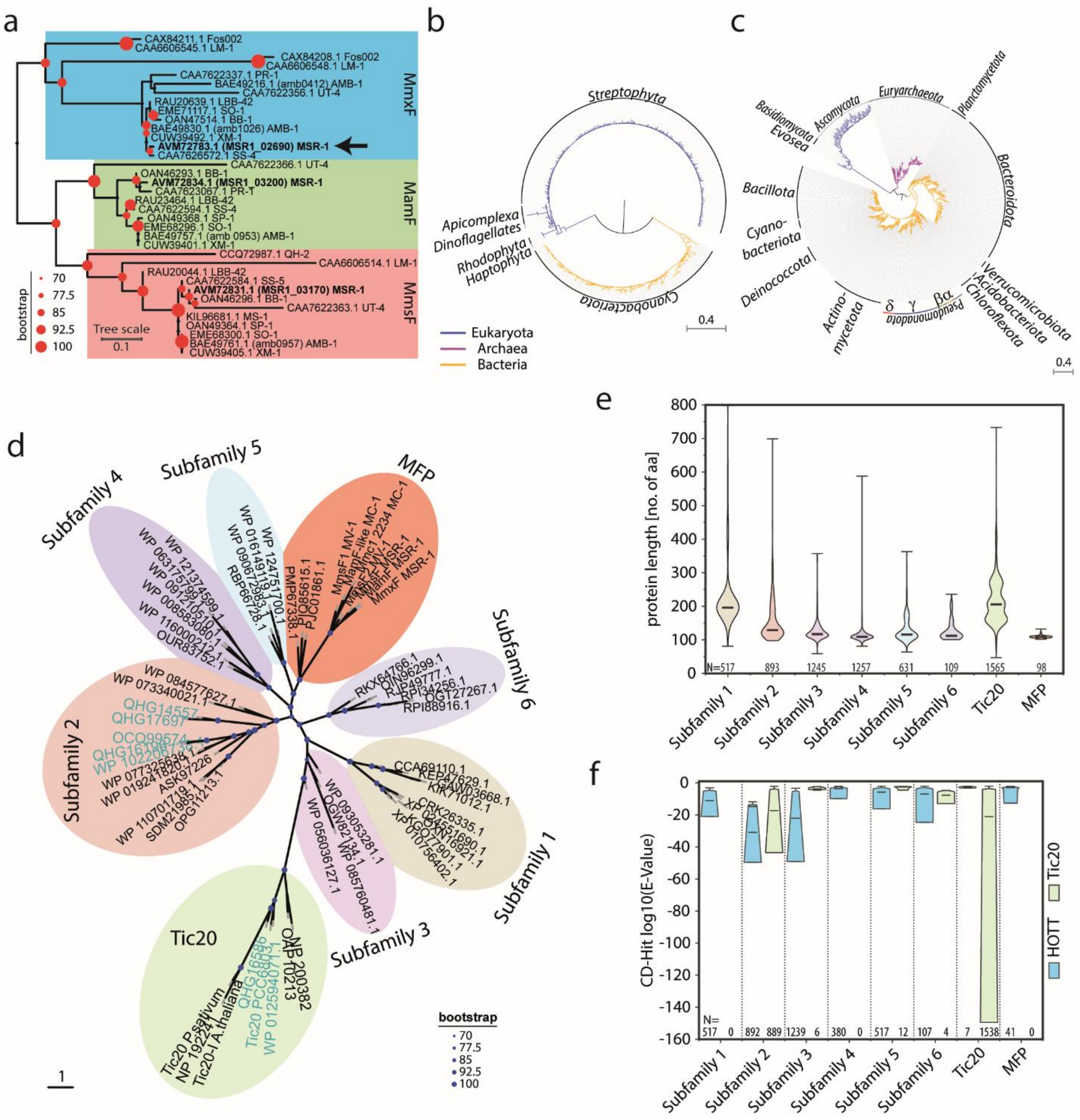

Supplementary Figure 1. MFPs are members of a common Tic20/HOTT protein superfamily, which are distributed in all domains of life. a A phylogenetic analysis of various MFPs reveals that one of the newly identified magnetosome proteins (MSR1_02690) is an ortholog of MmxF (amb1026) from *Magnetosprillum magneticum* AMB-1 [14]. The tree is based on 36 MFPs sequences from different magnetotactic bacteria inferred under the best-fitting LG+G4 substitution model. Grouped MmxF, MamF and MmsF homologs are shaded with blue, green, and red boxes, respectively. MSR-1 MFPs are highlighted in bold and MmxF is marked with an arrow. Red dots above branches are bootstrap values in percent, denoted in the legend. Scale bar represents expected substitutions per site. Accession numbers are listed in **Supplementary Data 1**. **b** A phylogenetic analysis reveals a narrow distribution of the Tic20 family. The phylogenetic tree is based on 16S/18S rRNA sequences from 297 organisms containing at least one Tic20 family protein. The tree was inferred under the best-fitting TIM3+F+R3 substitution model. Scale bar represents expected substitutions per site. **c** A phylogenetic analysis reveals the wide distribution of the HOTT family in all domains of life. The phylogenetic tree is based on 16S/18S rRNA sequences from 456 organisms containing at least one HOTT family protein. The tree was inferred under the best-fitting GTR+F+R10 substitution model. Scale bar represents expected substitutions per site. **d** Unrooted phylogenetic tree of the Tic20/HOTT superfamily inferred under the best-fitting mtInv+F+I+G4 substitution model. The MFPs and six other HOTT groups form distinct subfamilies within the HOTT family that is widely separated from the Tic20 family. HOTT subfamily 1 exclusively includes eukaryotic sequences whereas subfamilies 2–6 contain only prokaryotic representatives. Cyanobacteria (in teal) are the only organisms containing HOTT (subfamily 2) and Tic20 family proteins. Bootstrap values (blue dots) are denoted in the legend. Scale bar represents expected substitutions per site. Accession numbers are listed in **Supplementary Data 1**. **e** Comparison of protein length distributions (in aa) in the Tic20/HOTT superfamily using violin plots. Please note the short length of most HOTT family members in comparison to Tic20. The min, max, and mean values are indicated by bars. The number of analyzed proteins of each group [N] is given below. **f** Conserved domain (CD) search analysis of the Tic20/HOTT superfamily using violin plots. Almost all representatives of the HOTT Subfamily 2 retrieve significant E-values to both protein families, HOTT and Tic20. Only hits with E-values < than 10^{-3} are shown. The min, max, and mean values are indicated by bars. The number of proteins with hits to each protein domain [N] is given below. **a, d, e, f** Source data are provided as a Source Data file.

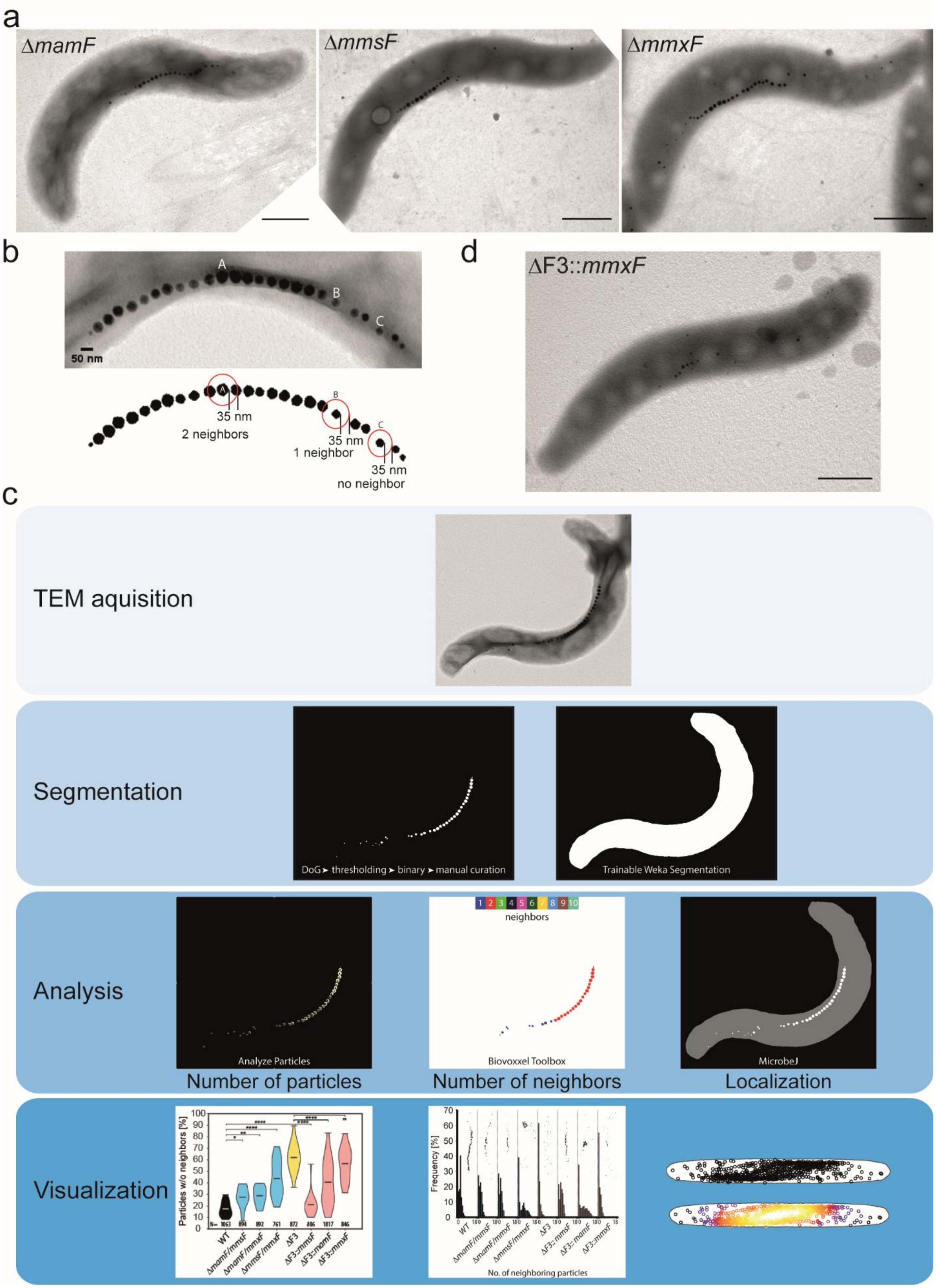
a
ΔmamF
ΔmmsF
ΔmmxF
b
A
B
C
50 nm
35 nm
2 neighbors
35 nm
1 neighbor
35 nm
no neighbor
d
ΔF3::mmxF
c
TEM aquisition
Segmentation
DoG ➤ thresholding ➤ binary ➤ manual curation
Trainable Weka Segmentation
Analysis
Analyze Particles
Number of particles
neighbors
Biovoxxel Toolbox
Number of neighbors
MicrobeJ
Localization
Visualization
Particles w/o neighbors [%]
Frequency [%]
No. of neighboring particles

Supplementary Figure 2. Characterization of MSR-1 deletion phenotypes using quantitative magnetosome neighbor analysis. a Representative TEM micrographs of MFP single deletions show WT-like phenotypes. (Scale bars: 500 nm). **b** MSR-1 WT magnetosomes with an average size of 35 nm are arranged in almost perfect chain-like structures in which most particles have exactly two neighboring particles within a distance of 35 nm (see particle A). Exceptions can be found at the end of chains where a wider particle spacing can be observed (e.g. particle B that has only one neighboring particle within 35 nm) or particle C that has no neighbor. In the absence of magnetosome chains the frequency of neighbor-less particles increases strongly while strains with aggregated magnetosomes frequently have neighbor numbers ≥ 3. **c** For quantitative magnetosome neighbor analysis images of different mutant strains were collected by TEM. Micrographs were subsequently segmented into magnetosomes and cell bodies. Segmented binary images were then used to analyze the overall number of magnetosomes per cell, the number of neighboring magnetosomes of each individual particle and the distribution of intracellular magnetosome localizations using Fiji plugins. Visualization of the results was performed using Fit-o-mat 0.752 [6], Prism 7 (GraphPad Prism Software Inc., San Diego, California), and MicrobeJ [7]. **d** Expression of *mmxF* in ΔF3 results in phenotypic heterogeneity (compare with ΔF3::*mmxF* TEM image in **Fig. 2c**) which may derive from an unbalanced production of MmxF or an impaired folding due to the use of the strong *mamG* promoter.

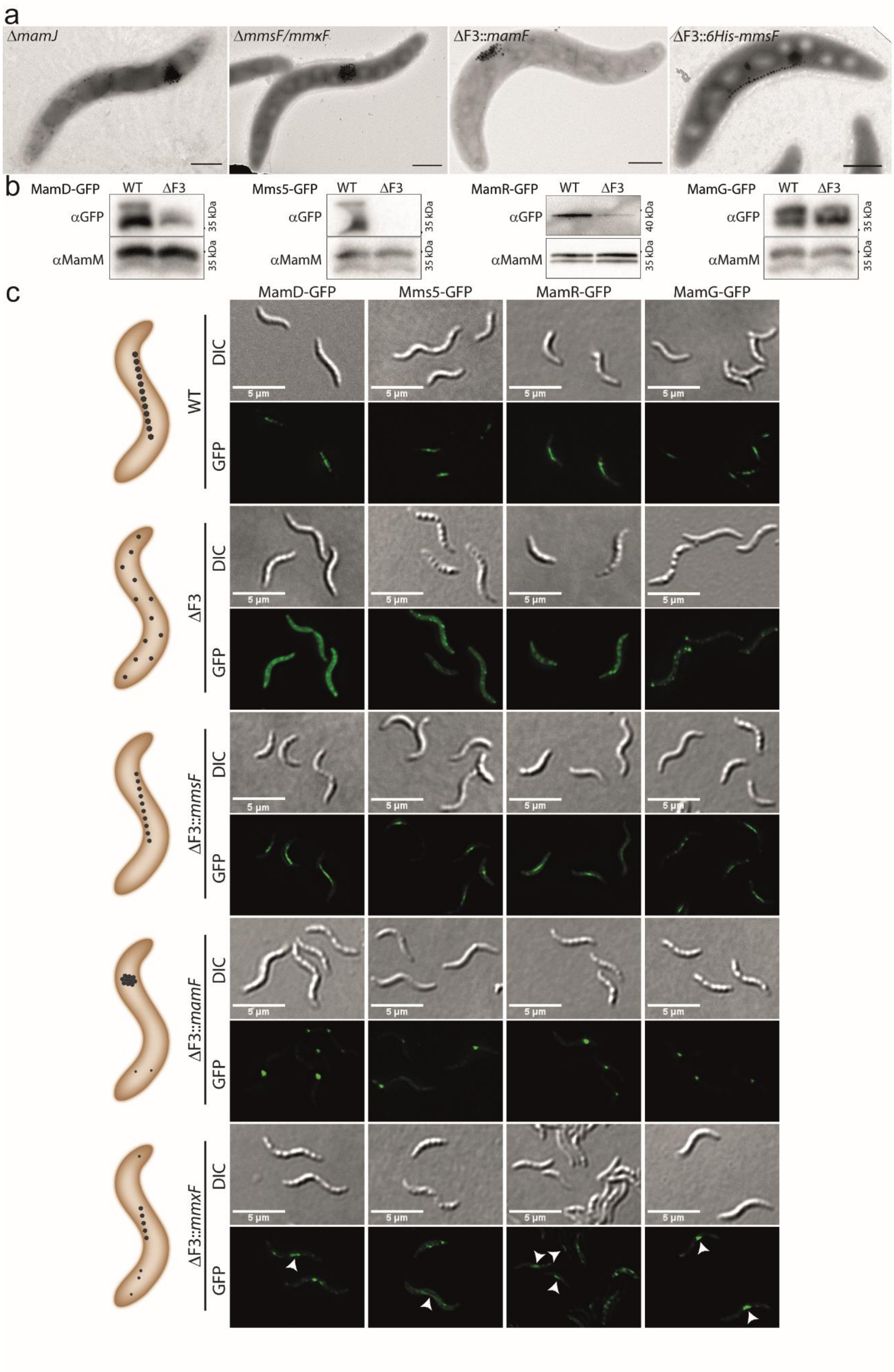

a
ΔmamJ
ΔmmsF/mmxF
ΔF3::mamF
ΔF3::6His-mmsF
b
MamD-GFP
WT
ΔF3
αGFP
αMamM
35 kDa
Mms5-GFP
MamR-GFP
40 kDa
MamG-GFP
c
MamD-GFP
Mms5-GFP
MamR-GFP
MamG-GFP
DIC
GFP
WT
ΔF3
ΔF3::mmsF
ΔF3::mamF
ΔF3::mmxF
5 μm

Supplementary Figure 3. Verification of the ΔF3 proteome analysis. a MSR-1 mutants expressing *mamF* as the only MFP gene phenocopy the *mamJ* deletion mutant whereas ΔF3 expressing a *6His-mmsF* construct phenocopies strain ΔF3::*mmsF* (compare **Fig. 2c**). Representative TEM micrographs of Δ*mamJ*, Δ*mmsF/mmxF*, ΔF3::*mamF,* and ΔF3::*mmsF*. (Scale bars: 500 nm). **b** Western blot analyses (αGFP) of magnetosome membrane fractions from WT and ΔF3 strains expressing *mamG-*, *mamD-*, *mms5-*, and *mamR-GFP* confirm a strong reduction of MamD, Mms5, and MamR in ΔF3 magnetosomes compared to the WT. In contrast, MamG-GFP is equally abundant in both strains. Source data are provided as a Source Data file. **c** Schematic phenotypic models and epifluorescence imaging of WT, ΔF3, and complemented ΔF3 strains producing MamD-, Mms5-, MamR-, or MamG-GFP fusion proteins reveals MM targeting of MamD-, Mms5-, and MamR-GFP by all three MFPs. In ΔF3, MamD-, MamR-, and partially Mms5-GFP show only a soluble localization whereas MamG-GFP exclusively localizes in a punctuate pattern. Arrowheads indicate cells with midcell chain-like fluorescence patterns in ΔF3::*mmxF*. DIC, differential interference contrast image. (Scale bars: 5 µm)

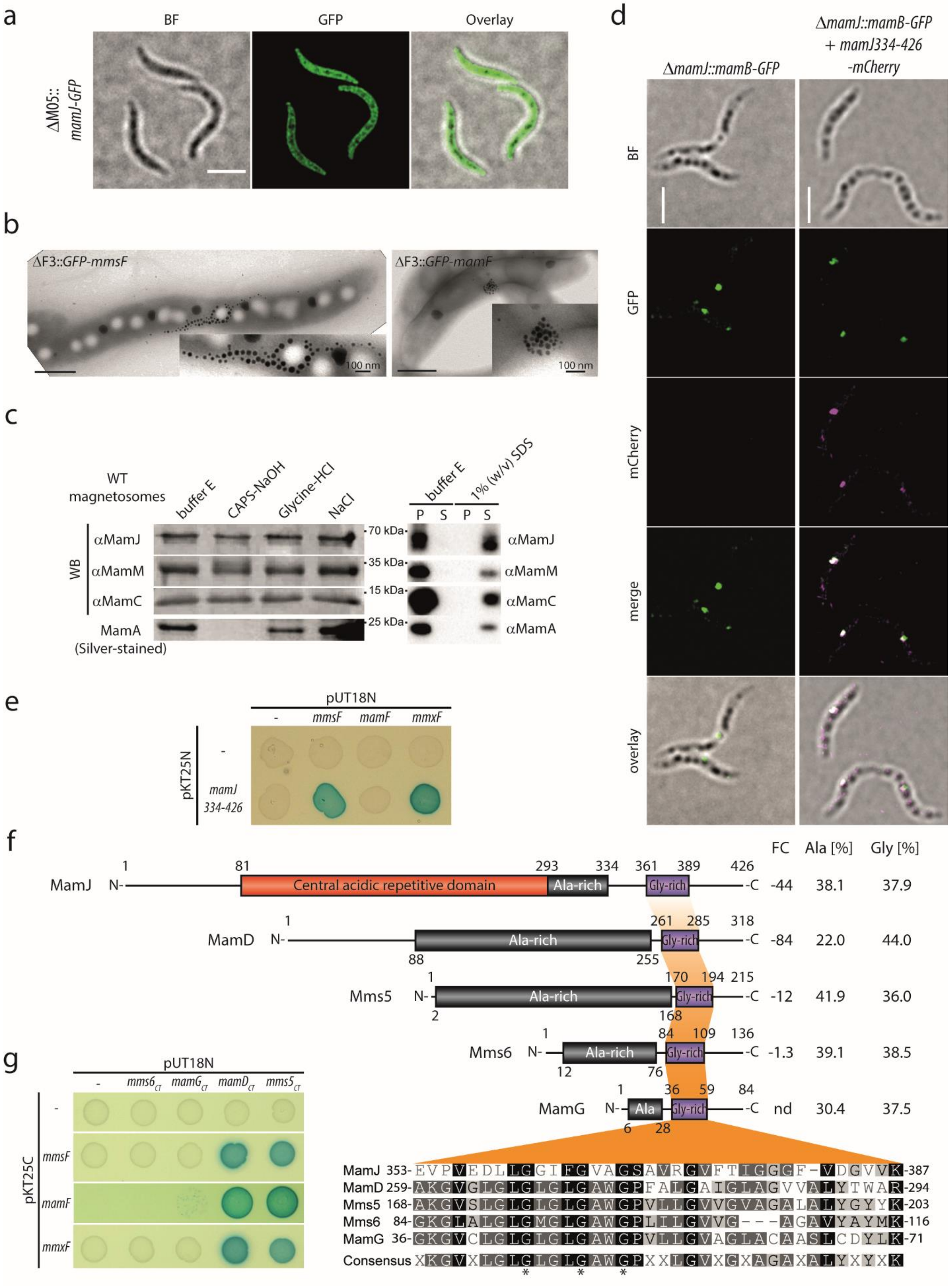
a
BF
GFP
Overlay
ΔM05::
mamJ-GFP
b
ΔF3::GFP-mmsF
100 nm
ΔF3::GFP-mamF
100 nm
c
WT
magnetosomes
buffer E
CAPS-NaOH
Glycine-HCl
NaCl
WB
αMamJ
αMamM
αMamC
MamA
(Silver-stained)
70 kDa
35 kDa
15 kDa
25 kDa
buffer E
1% (w/v) SDS
P S P S
αMamJ
αMamM
αMamC
αMamA
d
ΔmamJ::mamB-GFP
ΔmamJ::mamB-GFP
+ mamJ334-426
-mCherry
BF
GFP
mCherry
merge
overlay
e
pUT18N
-
mmsF
mamF
mmxF
pKT25N
-
mamJ
334-426
f
FC
Ala [%]
Gly [%]
MamJ
N-
1
81
Central acidic repetitive domain
293
Ala-rich
334
361
Gly-rich
389
426
-C
-44
38.1
37.9
MamD
N-
1
88
Ala-rich
255
261
Gly-rich
285
318
-C
-84
22.0
44.0
Mms5
N-
1
2
Ala-rich
168
170
Gly-rich
194
215
-C
-12
41.9
36.0
Mms6
N-
1
12
Ala-rich
76
84
Gly-rich
109
136
-C
-1.3
39.1
38.5
MamG
N-
1
6
Ala
28
36
Gly-rich
59
84
-C
nd
30.4
37.5
MamJ 353-EVPVEDLLGGIFGVAGSAVRGVFTIGGGF-VDGVVK-387
MamD 259-AKGVGLGLGLGLGAWGPFALGAIGLAGVVALYTWAR-294
Mms5 168-AKGVSLGLGLGLGAWGPVLLGVVGVAGALALYGYYK-203
Mms6 84-GKGLALGLGMGLGAWGPLILGVVG---AGAVYAYMK-116
MamG 36-GKGVCLGLGLGLGAWGPVLLGVAGLACAASLCDYLK-71
Consensus XKGVXLGLGLGLGAWGPXXLGVXGXAGAXALYXYXK
* * *
g
pUT18N
-
mms6CT
mamGCT
mamDCT
mms5CT
pKT25C
-
mmsF
mamF
mmxF

Supplementary Figure 4. Characterization of potential MFP substrate proteins. a 3D-Structured illumination microscopy of MamJ-GFP reveals a soluble localization pattern in the MSR-1 ΔM05 mutant that lacks all magnetosome genes. BF, bright field image. (Scale bars: 2 µm) **b** GFP-labeled MmsF and MamF are functional. Complementation of ΔF3 with *GFP-mmsF* or *GFP-mamF* results in similar phenotypes as complementation with unlabeled genes (compare with **Fig. 2c**). (Scale bars: 500 nm). **c** MamJ is resistant to prolonged treatments (20 h) with basic (0.1 M N-cyclohexyl-3-aminopropanesulfonic acid (CAPS), pH 11), acidic (0.1 M glycine, pH 2.5), or high salt (1 M NaCl, 10 mM Hepes, 1 mM EDTA, pH 7.4) buffer solutions. Magnetosome pellet fractions were loaded on SDS-PAGE and subsequently analyzed by Western blot (αMamJ, αMamM, αMamC) or silver staining (MamA). In an independent experiment, magnetosomes were incubated for one hour in 1% SDS as a positive control. The sample was fractionated into supernatant and magnetosome pellets by centrifugation. Trichloroacetic acid-precipitated supernatant and pellet fractions were loaded onto SDS-PAGE. Subsequent Western blot immunodetections of MamJ, MamM, and MamC indicate an integral membrane protein behavior for all three proteins as they could be only extracted by SDS. Contrarily, the MM associated protein MamA could be readily extracted by SDS and CAPS-buffer treatments. Buffer E treatments served as negative controls. Source data are provided as a Source Data file. **d** 3D-Structured illumination colocalization microscopy of MamB-GFP and MamJ334-426-mCherry in Δ*mamJ* indicate that residues 334-426 mediate MM targeting of MamJ (see also **Fig. 4**). Micrographs are maximum intensity projections. MamB-GFP is colored in green; MamJ-mCherry is colored in magenta; White-colored regions in the overlay indicate colocalization. BF, bright field image. (Scale bars: 2 µm) **e** Interaction analysis of MamJ using a BACTH assay based on reconstitution of CyaA adenylate cyclase activity in the *cya*$^{-}$ strain *E. coli* BTH101. Fusion of proteins to the fragmented catalytic CyaA domains T25 and T18 from *Bordetella pertussis* only confers cAMP-dependent expression of a *lacZ* reporter gene upon protein-protein interaction-mediated reconstitution of CyaA. *lacZ* expression is indicated by blue color formation and enhanced growth on X-Gal containing M63 maltose-mineral salts agar. The MamJ C-terminal domain interacts with MmsF and MmxF but not MamF in BACTH assays. Source data are provided as a Source Data file. **f** Domain structures of putative MFP substrates MamJ, MamD and Mms5 as well as the MamD-homologs MamG and Mms6 which are not targeted to the MM in an MFP-dependent manner (fold change (FC) of the proteomic analysis is indicated; nd, not detected). Alanine and glycine contents of the alanine- (black) and glycine-rich (purple) domains are given in %, respectively. Numbers above and below the proteins represent domain boundaries or protein lengths, respectively. Multiple aa sequence alignment of the glycine-rich domains. Amino acid residues are shaded using the Rasmol color scheme. A consensus motif is given below the alignment. The numbers at the beginning and end of each line indicate the position of the first and last amino acid of the respective protein within the alignment. Asterisks indicate the position of conserved glycine residues. **g** Interaction analysis of MamD, Mms5, MamG, Mms6 C-terminal domains using a BACTH assay based on reconstitution of CyaA adenylate cyclase activity in the *cya*$^{-}$ strain *E. coli* BTH101. BACTH assay showing the interaction between the C-terminal domains of MamD and Mms5 with MamF, MmsF, and MmxF. Note that the highly homologous proteins MamG and Mms6 do not interact with the MFPs. Source data are provided as a Source Data file.

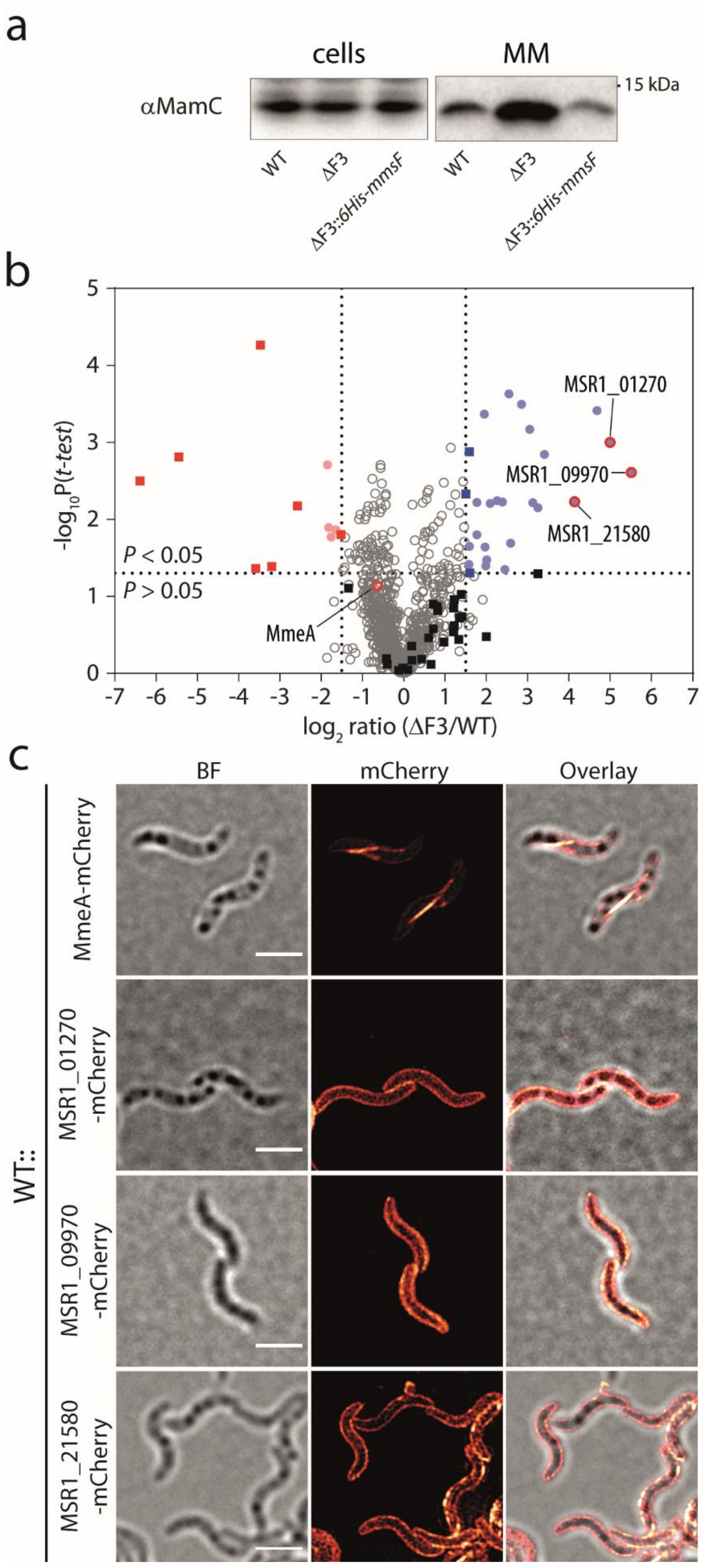
a
cells
MM
15 kDa
αMamC
WT
ΔF3
ΔF3::6His-mmsF
WT
ΔF3
ΔF3::6His-mmsF
b
MSR1_01270
MSR1_09970
MSR1_21580
P < 0.05
P > 0.05
MmeA
-log10P(t-test)
log2 ratio (ΔF3/WT)
c
BF
mCherry
Overlay
WT::
MmeA-mCherry
MSR1_01270 -mCherry
MSR1_09970 -mCherry
MSR1_21580 -mCherry

Supplementary Figure 5. Verification of the proteomic data a Western immunoblot analysis of the WT, ΔF3 and ΔF3::6His-*mmsF* strains using MamC antibody confirms the slight enrichment of MamC in magnetosome membrane fractions of ΔF3 while the cellular levels remained unaltered. Molecular weight standard is indicated. Source data are provided as a Source Data file. **b** Volcano plot showing the log_2-fold depletion (red) or enrichment (blue) of proteins in purified magnetosome fractions of ΔF3 compared to the WT as determined by LC-MS/MS. Circles represent non-MAI encoded proteins, non-significant proteins are depicted by grey or black symbols, respectively. Proteins with predicted N-terminal signal peptide sequences and high enrichment in ΔF3 used for mCherry-labelling are highlighted (red circles). MmeA, whose abundance remained unaffected in ΔF3 MM fractions and used as a reference for mCherry labelling is also highlighted. Coloring otherwise identical to **Fig. 3d**. (See also **Supplementary Data 4**. Source data are provided as a Source Data file.) **c** 3D-Structured illumination microscopy confirms that mCherry-labeled proteins (see **Supplementary Fig. 5b**) with potential secretory signal sequences are localized near the cell surface in the MSR-1 WT. However, except for the known magnetosome protein MmeA, no protein showed magnetosome chain localization. BF, bright field image. (Scale bars: 2 μM)

Supplementary Table 1. Strains used in this study.

Strain	Genotype/Description	Reference/Source
E. coli		
DH5α	Host for cloning: F$^-$ Φ80*lac*ZΔM15 Δ(*lac*ZYA-*arg*F) U169 *rec*A1 *end*A1 *hsd*R17 (r_k^-, m_k^+) *pho*A *sup*E44 *thi*-1 *gyr*A96 *rel*A1 λ$^-$	Invitrogen
WM3064	Donor strain for conjugation: *thrB1004 pro thi rpsL hsdS lacZ*ΔM15 RP4-1360 Δ*(araBAD)*567 Δ*dap*A1341::[*erm pir* (wt)]	William Metcalf at UIUC
BTH101	BACTH assay reporter strain: F$^-$ *cya-99 araD139 galE15 galK16 rpsL1* (Str^R) *hsdR2 mcrA1 mcrB1*	Euromedex
M. gryphiswaldense		
Wild type (WT)	MSR-1 R3/S1; Rif^R, Sm^R spontaneous mutant	[8]
WT::*mamD-GFP*	WT with *mamD-GFP* after Tn5 insertion, Tet^R	This study
WT::*mamR-GFP*	WT with *mamR-GFP* after Tn5 insertion, Tet^R	This study
WT::*mms5-GFP*	WT with *mms5-GFP* after Tn5 insertion, Tet^R	This study
WT::*mamG-GFP*	WT with *mamG-GFP* after Tn5 insertion, Tet^R	This study
WT::P$_{tet}$-*mmeA-mCherry*	WT with *mmeA-mCherry* after Tn5 insertion, Kan^R	This study
WT::*MSR-1_01270-mCherry*	WT with *MSR-1_01270-mCherry* after Tn7 insertion, Cam^R	This study
WT::*MSR-1_09970-mCherry*	WT with *MSR-1_09970-mCherry* after Tn7 insertion, Cam^R	This study
WT::*MSR-1_21580-mCherry*	WT with *MSR-1_21580-mCherry* after Tn7 insertion, Cam^R	This study
WT::*MSR-1_03900-mCherry*	WT with *MSR-1_03900-mCherry* after Tn7 insertion, Cam^R	This study
ΔM05	Non-magnetic mutant with large MAI operon deletions	[9]
ΔM05::*mamJ-GFP*	Δ*M05* with *mamJ-GFP* after Tn5 insertion, Tet^R	This study
Δ*mamJ*	*mamJ* deletion strain	[10]
Δ*mamJ::mamJ-GFP*	Δ*mamJ* with *mamJ-GFP* after Tn5 insertion, Tet^R	This study
Δ*mamJ::mamJ1-80-GFP*	Δ*mamJ* with *mamJ1-80-GFP* after Tn5 insertion, Tet^R	This study
Δ*mamJ::mamJ81-333-GFP*	Δ*mamJ* with *mamJ81-333-GFP* after Tn5 insertion, Tet^R	This study
Δ*mamJ::mamJ334-426-GFP*	Δ*mamJ* with *mamJ334-426-GFP* after Tn5 insertion, Tet^R	This study
Δ*mamJ::mamJΔ335-343-GFP*	Δ*mamJ* with *mamJΔ334-343-GFP* after Tn5 insertion, Tet^R	This study
Δ*mamJ::mamJΔ335-348-GFP*	Δ*mamJ* with *mamJΔ334-348-GFP* after Tn5 insertion, Tet^R	This study
Δ*mamJ::mamJΔ335-353-GFP*	Δ*mamJ* with *mamJΔ334-353-GFP* after Tn5 insertion, Tet^R	This study
Δ*mamJ::mamJΔ335-358-GFP*	Δ*mamJ* with *mamJΔ334-358-GFP* after Tn5 insertion, Tet^R	This study
Δ*mamJ::mamJΔ378-426-GFP*	Δ*mamJ* with *mamJΔ378-426-GFP* after Tn5 insertion, Tet^R	This study
Δ*mamJ::mamJΔ396-426-GFP*	Δ*mamJ* with *mamJΔ396-426-GFP* after Tn5 insertion, Tet^R	This study
Δ*mamJ::mamJΔ407-426-GFP*	Δ*mamJ* with *mamJΔ407-426-GFP* after Tn5 insertion, Tet^R	This study

Strain	Genotype/Description	Reference/Source
Δ*mamJ::mamJΔ417-426-GFP*	Δ*mamJ* with *mamJΔ417-426-GFP* after Tn5 insertion, Tet^R	This study
Δ*mamJ::mamB-GFP*	Δ*mamJ* with chromosomally encoded *mamB-GFP,* expressed from the native *mamB* locus	This study
Δ*mamJ::mamB-GFP::* P_{lac}*-mamJ334-426-mCherry*	Δ*mamJ* with chromosomally encoded *mamB-GFP*, expressed from the native *mamB* locus and *mamJ334-426-mCherry* inserted into the Tn7 locus	This study
Δ*mamF*	*mamF* deletion strain	[11]
Δ*mmsF*	*mmsF* deletion strain	[11]
Δ*mmxF*	*mmxF* deletion strain	This study
Δ*mamF/mmsF*	*mamF*, *mmsF* double deletion strain	[11]
Δ*mamF/mmxF*	*mamF*, *mmxF* double deletion strain	This study
Δ*mmsF/mmxF*	*mmsF*, *mmxF* double deletion strain	This study
Δ*mmsF/mmxF::mamJ334-426-GFP*	Δ*mmsF/mmxF* mamJ334-426 after Tn5 insertion, Tet^R	
Δ*F3*	*mamF*, *mmsF*, *mmxF* triple deletion strain	This study
Δ*F3::mamF*	Tn5 mediated complementation of Δ*F3* with *mamF*, Tet^R	This study
Δ*F3::mmsF*	Tn5 mediated complementation of Δ*F3* with *mmsF*, Tet^R	This study
Δ*F3::mmxF*	Tn5 mediated complementation of Δ*F3* with *mmxF*, Tet^R	This study
Δ*F3::*6*His-mmsF*	*ΔF3* with *6His-mmsF* after Tn5 insertion, Kan^R	This study
Δ*F3::*P_{tet}*-mCherry-mamK*	Δ*F3* with inducible *mCherry-mamK* after Tn5 insertion, Kan^R	This study
Δ*F3::*P_{tet}*-mScarlet-I-mamY*	Δ*F3* with inducible *mScarlet-I-mamY* after Tn5 insertion, Kan^R	This study
Δ*F3*ΔmamJ*::mamJ-GFP*	Δ*F3*ΔmamJ with *mamJ-GFP* after Tn5 insertion, Tet^R	This study
Δ*F3*::*GFP-mamF*	Δ*F3* with *GFP-mamF* after Tn5 insertion, Tet^R	This study
Δ*F3*::*GFP-mmsF*	Δ*F3* with *GFP-mmsF* after Tn5 insertion, Tet^R	This study
Δ*F3*::*mamD-GFP*	Δ*F3* with *mamD-GFP* after Tn5 insertion, Tet^R	This study
Δ*F3*::*mamR-GFP*	Δ*F3* with *mamR-GFP* after Tn5 insertion, Tet^R	This study
Δ*F3*::*mms5-GFP*	Δ*F3* with *mms5-GFP* after Tn5 insertion, Tet^R	This study
Δ*F3*::*mamG-GFP*	Δ*F3* with *mamG-GFP* after Tn5 insertion, Tet^R	This study
Δ*F3*::*mamD-GFP::mmsF*	Δ*F3* with *mamD-GFP* and *mmsF* after Tn5 insertion, Kan^R, Tet^R	This study
Δ*F3*::*mamR-GFP::mmsF*	Δ*F3* with *mamR-GFP* and *mmsF* after Tn5 insertion, Kan^R, Tet^R	This study
Δ*F3*::*mms5-GFP::mmsF*	Δ*F3* with *mms5-GFP* and *mmsF* after Tn5 insertion, Kan^R, Tet^R	This study
Δ*F3*::*mamG-GFP::mmsF*	Δ*F3* with *mamG-GPF* and *mmsF* after Tn5 insertion, Kan^R, Tet^R	This study
Δ*F3*::*mamD-GFP::mamF*	Δ*F3* with *mamD-GFP* and *mamF* after Tn5 insertion, Kan^R, Tet^R	This study
Δ*F3*::*mamR-GFP::mamF*	Δ*F3* with *mamR-GFP* and *mamF* after Tn5 insertion, Kan^R, Tet^R	This study
Δ*F3*::*mms5-GFP::mamF*	Δ*F3* with *mms5-GFP* and *mamF* after Tn5 insertion, Kan^R, Tet^R	This study
Δ*F3*::*mamG-GFP::mamF*	Δ*F3* with *mamG-GPF* and *mamF* after Tn5 insertion, Kan^R, Tet^R	This study
Δ*F3*::*mamD-GFP::mmxF*	Δ*F3* with *mamD-GFP* and *mmxF* after Tn5 insertion, Kan^R, Tet^R	This study
Δ*F3*::*mamR-GFP::mmxF*	Δ*F3* with *mamR-GFP* and *mmxF* after Tn5 insertion, Kan^R, Tet^R	This study

Strain	Genotype/Description	Reference/Source
Δ*F3*::*mms5-GFP::mmxF*	Δ*F3* with *mms5-GFP* and *mmxF* after Tn5 insertion, Kan^R, Tet^R	This study
Δ*F3*::*mamG-GFP::mmxF*	Δ*F3* with *mamG-GPF* and *mmxF* after Tn5 insertion, Kan^R, Tet^R	This study
Δ*F3*::*mmsF* $^{D34N,\ D36N,\ D37N,\ E38N}$	Δ*F3* with *mmsF* $^{D34N,\ D36N,\ D37N,\ E38N}$ after Tn5 insertion, Tet^R	This study
Δ*F3*::*mmsF* $^{D34N,\ R35Q,\ D36N,\ D37N,\ E38N}$	Δ*F3* with *mmsF* $^{D34N,\ R35Q,\ D36N,\ D37N,\ E38N}$ after Tn5 insertion, Tet^R	This study
Δ*mamKY*	*mamK, mamY* double deletion strain	[12]
Δ*F3*Δ*mms5*Δ*mamK*Δ*mamY::GFP-mmsF*	*mamF*, *mmsF*, *mmxF*, *mms5*, *mamK*, *mamY* codeletion strain; ΔF3Δ*mms5*Δ*mamK*Δ*mamY* with *GFP-mmsF* after Tn5 insertion, Tet^R	This study
ΔA13Δ*mmxF*Δ*mms5*	*mamGFDC, mms5*, *mms6*, *mamXY* codeletion strain	[13]
ΔA13Δ*mmxF*Δ*mms5*Δ*mamR*	*mamGFDC, mms5*, *mms6*, *mamXY*, *MamR* codeletion strain	This study
ΔA13Δ*mmxF*Δ*mms5*Δ*mamR*::*mmsF*	ΔA13Δ*mmxF*Δ*mms5*Δ*mamR* with *mmsF* after Tn5 insertion, Tet^R	This study

Supplementary Table 2. Plasmids used in this study.

Plasmid	Description	Reference/Source
pUT18C	BACTH vector designed to express a given polypeptide fused in frame at its N-terminal end with T18 fragment; *ColE1* ori; Amp^R	[14]
pUT18N	BACTH vector designed to express a given polypeptide fused in frame at its C-terminal end with T18 fragment; *ColE1* ori; Amp^R	[14]
pKT25C	BACTH vector designed to express a given polypeptide fused in frame at its N-terminal end with T25 fragment; *p15A* ori; Kan^R	[14]
pKT25N	BACTH vector designed to express a given polypeptide fused in frame at its C-terminal end with T25 fragment; *p15A* ori; Kan^R	[14]
pUT18C-*zip*; pKT25-*zip*	Derivatives of pUT18C and pKT25 with a 114 bp DNA fragment encoding for a leucine zipper (positive control for BACTH)	[14]
pUT18C-*mamJ*	BACTH plasmid coding for T18-MamJ	[12]
pKT25C-*mamK*	BACTH plasmid coding for T25-MamK	[12]
pKT25C-*mamY*	BACTH plasmid coding for T25-MamY	[12]
pKT25N-*mamJ*334-426	BACTH plasmid coding for MamJ334-426-T25	This study
pUT18N-*mmsF*	BACTH plasmid coding for MmsF-T18	This study
pKT25C-*mmsF*	BACTH plasmid coding for T25-MmsF	This study
pUT18N-*mmxF*	BACTH plasmid coding for MmxF-T18	This study
pKT25C-*mmxF*	BACTH plasmid coding for T25-MmxF	This study
pUT18N-*mamF*	BACTH plasmid coding for MamF-T18	This study
pKT25C-*mamF*	BACTH plasmid coding for T25-MamF	This study
pUT18N-$mms6_{CT}$	BACTH plasmid coding for Mms6-T18	This study
pUT18N-$mamG_{CT}$	BACTH plasmid coding for MamG-T18	This study
pUT18N-$mamD_{CT}$	BACTH plasmid coding for MamD-T18	This study
pUT18N-$mms5_{CT}$	BACTH plasmid coding for Mms5-T18	This study
pBAM-Tet-*mamD-GFP*	*R6K* ori, Tn5, Amp^R, Tet^R, P_{mamG}-*mamD*-GFP	[15]
pBAM-Tet-*mamR-GFP*	*R6K* ori, Tn5, Amp^R, Tet^R, P_{mamG}-*mamR*-GFP	This study
pBAM-Tet-*mms5-GFP*	*R6K* ori, Tn5, Amp^R, Tet^R, P_{mamG}-*mms5*-GFP	This study
pBAM-Tet-*mamG-GFP*	*R6K* ori, Tn5, Amp^R, Tet^R, P_{mamG}-*mamG*-GFP	This study
pBAM-Tet-*mamJ-GFP*	*R6K* ori, Tn5, Amp^R, Tet^R, P_{mamG}-*mamJ*-GFP	[16]
pBAM-Tet-*mamJ1-80-GFP*	*R6K* ori, Tn5, Amp^R, Tet^R, P_{mamG}-*mamJ1-80-GFP*	This study
pBAM-Tet-*mamJ81-333-GFP*	*R6K* ori, Tn5, Amp^R, Tet^R, P_{mamG}-*mamJ81-333-GFP*	This study
pBAM-Tet-*mamJ335-426-GFP*	*R6K* ori, Tn5, Amp^R, Tet^R, P_{mamG}-*mamJ335-426-GFP*	This study
pBAM-Tet-*mamJΔ335-343-GFP*	*R6K* ori, Tn5, Amp^R, Tet^R, P_{mamG}-*mamJΔ335-343-GFP*	This study
pBAM-Tet-*mamJΔ335-348-GFP*	*R6K* ori, Tn5, Amp^R, Tet^R, P_{mamG}-*mamJΔ335-348-GFP*	This study
pBAM-Tet-*mamJΔ335-353-GFP*	*R6K* ori, Tn5, Amp^R, Tet^R, P_{mamG}-*mamJΔ335-353-GFP*	This study

Plasmid	Description	Reference/Source
pBAM-Tet-*mamJΔ334-359-GFP*	*R6K* ori, Tn5, Amp^{R}, Tet^{R}, P$_{mamG}$-*mamJΔ335-358-GFP*	This study
pBAM-Tet-*mamJΔ378-426-GFP*	*R6K* ori, Tn5, Amp^{R}, Tet^{R}, P$_{mamG}$-*mamJΔ378-426-GFP*	This study
pBAM-Tet-*mamJΔ396-426-GFP*	*R6K* ori, Tn5, Amp^{R}, Tet^{R}, P$_{mamG}$-*mamJΔ396-426-GFP*	This study
pBAM-Tet-*mamJΔ407-426-GFP*	*R6K* ori, Tn5, Amp^{R}, Tet^{R}, P$_{mamG}$-*mamJΔ407-426-GFP*	This study
pBAM-Tet-*mamJ*Δ417-426-GFP	*R6K* ori, Tn5, Amp^{R}, Tet^{R}, P$_{mamG}$-*mamJΔ417-426-GFP*	This study
pBAM-Tet-GFP-*mmsF*	*R6K* ori, Tn5, Amp^{R}, Tet^{R}, P$_{mamG}$-*GFP-mmsF*	This study
pBAM-Tet-GFP-*mamF*	*R6K* ori, Tn5, Amp^{R}, Tet^{R}, P$_{mamG}$-*GFP-mamF*	This study
pBAM-Tet-*mmsF*	*R6K* ori, Tn5, Amp^{R}, Tet^{R}, P$_{mamG}$-*mmsF*	This study
pBAM-Tet-*mamF*	*R6K* ori, Tn5, Amp^{R}, Tet^{R}, P$_{mamG}$-*mamF*	This study
pBAM-Tet-*mmxF*	*R6K* ori, Tn5, Amp^{R}, Tet^{R}, P$_{mamG}$-*mmxF*	This study
pBAM-Tet-*mmsF*$^{D34N, D36N, D37N, E38N}$	*R6K* ori, Tn5, Amp^{R}, Tet^{R}, P$_{mamG}$-*mmsF*	This study
pBAM-Tet-*mmsF*$^{D34N, R35Q, D36N, D37N, E38N}$	*R6K* ori, Tn5, Amp^{R}, Tet^{R}, P$_{mamG}$-*mmsF*	This study
pBAM1-*mmsF*	*R6K* ori, Tn5, Amp^{R}, Kan^{R}, P$_{mamG}$-*mmsF*	This study
pBAM1-*mamF*	*R6K* ori, Tn5, Amp^{R}, Kan^{R}, P$_{mamG}$-*mamF*	This study
pBAM1-*mmxF*	*R6K* ori, Tn5, Amp^{R}, Kan^{R}, P$_{mamG}$-*mmxF*	This study
pBAM2-His$_{6}$-*mmsF*	*p15A* ori, Tn5, Amp^{R}, Kan^{R} P$_{mamG}$-*6His-mmsF*	This study
pBAM2-MSR-1_09970-mCherry	*p15A* ori, Tn7, Amp^{R}, Cam^{R}, P$_{G}$-*MSR-1_09970-mCherry*	This study
pBAM2-MSR-1_01270-mCherry	*p15A* ori, Tn7, Amp^{R}, Cam^{R}, P$_{G}$-*MSR-1_01270-mCherry*	This study
pBAM2-MSR-1_21580-mCherry	*p15A* ori, Tn7, Amp^{R}, Cam^{R}, P$_{G}$-*MSR-1_21580-mCherry*	This study
pBAM2-MSR-1_03900-mCherry	*p15A* ori, Tn7, Amp^{R}, Cam^{R}, P$_{G}$-*MSR-1_03900-mCherry*	This study
pBAM2-*mamJ334-426*-mCherry	*p15A* ori, Tn7, Amp^{R}, Cam^{R}, P$_{lac}$-*mamJ334-426-mCherry*	This study
pBAM160-*mamA*-GFP	*R6K* ori, Tn5, Amp^{R}, Kan^{R}, P$_{tet}$-*mamA-GFP*	[17]
pBAM160-mCherry-*mamK*	*R6K* ori, Tn5, Amp^{R}, Kan^{R}, P$_{tet}$-*mCherry-mamK*	This study
pBAM160-*mmeA-mCherry*	*R6K* ori, Tn5, Amp^{R}, Kan^{R}, P$_{tet}$-*mmeA-mCherry*	This study
pBAM160-mScarlet-I-*mamY*	*R6K* ori, Tn5, Amp^{R}, Kan^{R}, P$_{tet}$-*mScarlet-I-mamY*	This study
pORFM-GalK-MCS	universal in-frame deletion/in-frame fusion vector for GalK counterselection; *npt galK* Tet^{R} *mobRK2*	[18]
pOR-Δ*mamR*	pORFM-GalK-MCS with Δ*mamR* deletion construct	This study
pOR-Δ*mamJ*	pORFM-GalK-MCS with Δ*mamJ* deletion construct	This study
pOR-*mamB-GFP*	pORFM-GalK-MCS with *mamB-GFP* insertion construct	This study

Supplementary Table 3. PCR Primers used in this study.

Oligonucleotides	Sequence	Source
dmmxF_do_for	CCGAAGCCGTCTGATGCTGAACGGATCCGCCGCGCC	This study
dmmxF_do_rev	CATCATCGGCAAGGAGGCGCAGA	This study
dmmxF_up_rev	GGATCCGTTCAGCATCAGACGGCTTCGGCCGCAGC	This study
dmmxF_up_for	CCGCAGCTCCCGCAGCTGGC	This study
MamR_Kpn_for	GGTACCATGATTTGGACAGCAGTGATC	This study
MamR-GFP_SacI_rev	GAGCTCTCACTTATACAGCTCGTCCATGC	This study
MmsF_Kpn_for	GGTACCATGGTTGAAGCAATCCTTCG	This study
MamF_Sac_rev	GAGCTCTCAGATCAGGGCGACTACAT	This study
MmeA_NdeI_for	CATATGGCCCTGAAGACGACCCATGC	This study
MmeAoStop_SacI_rev	GAGCTCGCGAACGTAGACCTGCACCT	This study
mms6_C-term2_for	AGGCTGTCGGCGGCACCATC	This study
mms6_C-term2_rev	GGGACAGCGCGTCGCGCAG	This study
mamG_C-term_for	AGGCTGTCGGCAGCACCTTG	This study
mamG_C-term_rev	GAGCAGGCTCGGCGGAGGC	This study
mamD_C-term2_for	AGGCCGCTGGCAGCGCCATG	This study
mamD_C-term2_rev	GTTCCTCGCCGACAGCCGCCAGAA	This study
mms5_C-term2_for	AGACCAGCAGCAGCGCCATGCT	This study
mms5_C-term2_rev	GGACGGCTTCGGCCGCAGC	This study
MmsF_for	AGGTTGAAGCAATCCTTCGG	This study
MmsF_rev	GGATCCGGTCGGCCACCCA	This study
MamF_for	AGGCCGAGACTATTTTGATC	This study
MamF_rev	GGATCAGGGCGACTACATG	This study
MmxF_for	AGATCGCACAGACTGTCGGG	This study
MmxF_rev	GGATGCGGTCGGCGATATA	This study
MamY_oSTop_Kpn_rev	GATCGGTACCCGCATCGGAGATGGGGGTT	This study
MamY_Nde_for	CGTACATATGTTGATGAACTTTGTCAACAATG	This study
mamJ_1-80.FOR	GTAACATATGGCAAAAAACCGGCGTGATCGC	This study
mamJ_335-392.FOR	GCCTCATATGACCCGCCAGCCTAACAAGAT	This study
mamJ_335-392.REV	GCTGGGTACCTTTATTCTTATCTTCAGCATCACAT	This study
mamJ_1-80.REV	ACGAGGTACCGTCCTGGGAACGAATGGG	This study
MamJ-E294_NdeI_for	GATCAGCATATGGAGAGCGTTGCATCAGCG	This study
MamJ-E333_KpnI_rev	GCTACGGGTACCTTCGACCGCCACAGCAAC	This study
MamJ-A334_NdeI_for	GATCAGCATATGGCCACCCGCCAGCCTAAC	This study
MamJ-F364_KpnI_rev	GCTACGGGTACCGAAAATCCCCCCCAGAAGGT	This study
MamJ-G365_NdeI_for	GATCAGCATATGGGCGTCGCCGGATCGGCG	This study
MamJ-N395_KpnI_rev	GCTACGGGTACCATTGCTTCCAACGAGGCGGCCTCC	This study
MamJ-V396_NdeI_for	GATCAGCATATGGTGGTCGCCGGGACGCGC	This study
MamJ-CAR_for	GATCAGCATATGCCTGTGCCGGTTGCCGATC	This study
MamJ-CAR_rev	GCTACGGGTACCTTCGACCGCCACAGCAAC	This study
MamJ1-80_NdeI_rev	GCTGACATATGGTCCTGGGAACGAATGGG	This study
MamJ344S_for	TCAGTTAAGAAGCGCGCCC	This study
MamJ349A_for	GCCCCGGTTCAGGAAGTTC	This study
MamJ354V_for	GTTCCCGTGGAAGACCTTCTG	This study
MamJ359L_for	CTTCTGGGGGGGATTTTCG	This study
Kpn-10G_for	GGTACCGGAGGCGGAGGCG	This study
MamJ416S_rev	CGATGAACAACTACCGCAACTTACCTC	This study
MamJ406T_rev	AGTTTGCGCCAACCGGCGC	This study
MamJ386V_rev	GACCACTCCATCGACGAATCCG	This study

Oligonucleotides	Sequence	Source
MamJ376T_rev	AGTGAACACACCCCGCACCG	This study
6His-MmsF-NdeI_for	AATTCATATGCATCACCACCATCATCACGTTGAAGCAATCCTT CGGAGC	This study
6His-MamF-NdeI_for	AATTCATATGCATCACCACCATCATCACGCCGAGACTATTTTG ATC	This study
MmsF_SphI_rev	TAAGGCATGCTCAGATCCGGTCGGCCACCCA	This study
MamF_SphI_rev	TAAGGCATGCTCAGATCAGGGCGACTACATG	This study
MamJS410_SmaI_rev	CCCGGGCACTTACCTCGATAGTTTGCGC	This study
MamJA334_for	AGGCCACCCGCCAGCCTAAC	This study
MamJ_rev	GTTTATTCTTATCTTCAGCATCACATTTCG	This study
Mms5_SacI_rev	GAGCTCTCAGACGGCTTCGGCCGC	This study
Mms5_KpnI_for	GGTACCATGGCTGGTGGGACCGCG	This study
MmxF_SacI_rev	GAGCTCTCAGATGCGGTCGGCGAT	This study
MmxF_KpnI_for	GGTACCATGATCGCACAGACTGTCG	This study
MamF_KpnI_for	GGTACCATGGCCGAGACTATTTTG	This study
MamF_SacI_rev	GAGCTCTCAGATCAGGGCGACTAC	This study
MmsF_SacI_rev	GAGCTCTCAGATCCGGTCGGCCAC	This study
MmsF_KpnI_for	GGTACCATGGTTGAAGCAATCCTTCG	This study

Supplementary Table 4. Media and buffers used in this study.

Component	Final concentration
MSR-1 flask standard medium (FSM) pH 7	
HEPES	10 mM
Potassium lactate	12 mM
$NaNO_3$	4 mM
KH_2PO_4	0.74 mM
$MgSO_4 \cdot 7\ H_2O$	0.60 mM
Fe(III)-citrate	50 µM
Yeast extract	0.01% (w/v)
Soybean peptone	0.3% (w/v)
MSR-1 soft agar pH 7	
HEPES	10 mM
Potassium lactate	1.5 mM
$NaNO_3$	4 mM
KH_2PO_4	0.74 mM
$MgSO_4 \cdot 7\ H_2O$	0.60 mM
Fe(III)-citrate	50 µM
Yeast extract	0.01% (w/v)
Soybean peptone	0.3% (w/v)
Agar	0.2% agar (w/v)
Agar for Bacterial two-hybrid assays	
X-Gal	40 µg mL^{-1}
IPTG	0.5 mM
Amicillin	100 µg mL^{-1}
Kanamycin	50 µg mL^{-1}
LB medium for Bacterial two-hybrid assays supplemented with	
IPTG	0.5 mM
Amicillin	100 µg mL^{-1}
Kanamycin	50 µg mL^{-1}
M 63 Agar for Bacterial two-hybrid assays	
X-Gal	40 µg mL^{-1}
IPTG	0.5 mM
Amicillin	50 µg mL^{-1}
Kanamycin	25 µg mL^{-1}
Maltose	0.2% (w/v)
Buffer W pH 7.4	
HEPES	20 mM
EDTA	5 mM
Buffer R pH 7.4	
HEPES	50 mM
EDTA	1 mM
PMSF	0.1 mM
Buffer E pH 7.4	
HEPES	10 mM
EDTA	1 mM
Buffer S pH 7.4	
HEPES	10 mM
EDTA	1 mM
NaCl	200 mM

Component	Final concentration
5x SDS sample buffer pH 6.8	
SDS	10% (w/v)
β-mercaptoethanol	25% (v/v)
Glycerol	25% (v/v)
Bromophenol blue	0.05%
Tris/HCl	0.3 M
2x SDS sample buffer pH 6.8	
SDS	0.2% (w/v)
DTT	200 mM
Glycerol	20% (v/v)
Bromophenol blue	0.2%
Tris	100 mM
Coomassie staining solution	
Methanol	50% (v/v)
Acetic acid	10% (v/v)
Coomassie R-250	0.1% (w/v)
Coomassie washing solution	
Methanol	10% (v/v)
Acetic acid	7% (v/v)
Silver-stain fixing solution	
Methanol	40% (v/v)
Formaldehyde	36.5% (v/v) of 37% (w/v)
Silver-stain incubation solution	
Thiosulfate	0.02% (w/v)
Silver-stain impregnation solution	
$AgNO_3$	0.1% (w/v)
Silver-stain developing solution	
$Na_2S_2O_3$	0.02% (v/v) of 0.02% (w/v)
Na_2CO_3	3% (w/v)
Formaldehyde	0.135% (v/v) of 37% (w/v)
Silver-stain stopping solution	
EDTA	1.86% (w/v)
Buffer E for protein complex purification supplemented with	
NaCl	50 mM
LMNG	0.1% (w/v)
Buffer E for protein complex purification supplemented with	
NaCl	50 mM
LMNG	0.1% (w/v)
Extraction buffers	
- $NaCO_3$ pH 11.3	100 mM
- Buffer E supplemented with SDS	1%
- CAPS-NaOH pH 11	0.1 M
- Glycine-HCl pH 2.5	0.1 M
- HEPES<br>EDTA pH7.4<br>NaCl	10 mM<br>1 mM<br>1M
Precipitation buffer	
Acetone	90% (v/v)
TCA	10% (v/v)

Component	Final concentration
NaCl	10 mM
Buffer 1 pH 7.4	
HEPES	10 mM
EDTA	1 mM
NaCl	50 mM
PMSF	0.1 mM
Triton X-100	0.5% (v/v)
Buffer 2 pH 7.4	
HEPES	10 mM
EDTA	1 mM
NaCl	50 mM
PMSF	0.1 mM
Triton X-100	0.1% (v/v)
Buffer A	
Acetic acid	0.1% (v/v)
Buffer B	
Acetic acid	0.1% (v/v)
Acetonitrile	99.9% (v/v)

Supplementary References

1. Scheffel, A., Gärdes, A., Grünberg, K., Wanner, G. & Schüler, D. The major magnetosome proteins MamGFDC are not essential for magnetite biomineralization in *Magnetospirillum gryphiswaldense* but regulate the size of magnetosome crystals. *J. Bacteriol.* **190,** 377–386, (2008).
2. Lohße, A. *et al.* Functional analysis of the magnetosome island in *Magnetospirillum gryphiswaldense*: the *mamAB* operon is sufficient for magnetite biomineralization. *PLoS One* **6,** e25561, (2011).
3. Raschdorf, O., Müller, F. D., Pósfai, M., Plitzko, J. M. & Schüler, D. The magnetosome proteins MamX, MamZ and MamH are involved in redox control of magnetite biomineralization in *Magnetospirillum gryphiswaldense*. *Mol. Microbiol.* **89,** 872–886, (2013).
4. Almagro Armenteros, J. J. *et al.* SignalP 5.0 improves signal peptide predictions using deep neural networks. *Nat. Biotechnol.* **37,** 420–423, (2019).
5. Richter, M. *et al.* Comparative genome analysis of four magnetotactic bacteria reveals a complex set of group-specific genes implicated in magnetosome biomineralization and function. *J. Bacteriol.* **189,** 4899–4910, (2007).
6. Möglich, A. An Open-Source, Cross-Platform Resource for Nonlinear Least-Squares Curve Fitting. *J. Chem. Educ.* **95,** 2273–2278 (2018).
7. Ducret, A., Quardokus, E. M. & Brun, Y. V. MicrobeJ, a tool for high throughput bacterial cell detection and quantitative analysis. *Nat. Microbiol.* **1,** 16077 (2016).
8. Schultheiss, D., Kube, M. & Schüler, D. Inactivation of the flagellin gene *flaA* in *Magnetospirillum gryphiswaldense* results in nonmagnetotactic mutants lacking flagellar filaments. *Appl. Environ. Microbiol.* **70,** 3624–3631, (2004).
9. Dziuba, M. V., Zwiener, T., Uebe, R. & Schüler, D. Single-step transfer of biosynthetic operons endows a non-magnetotactic Magnetospirillum strain from wetland with magnetosome biosynthesis. *Environ. Microbiol.* **22,** 1603–1618, (2020).
10. Scheffel, A. *et al.* An acidic protein aligns magnetosomes along a filamentous structure in magnetotactic bacteria. *Nature* **440,** 110–114 (2006).
11. Lohße, A. *et al.* Genetic dissection of the *mamAB* and *mms6* operons reveals a gene set essential for magnetosome biogenesis in *Magnetospirillum gryphiswaldense*. *J. Bacteriol.* **196,** 2658–2669, (2014).
12. Toro-Nahuelpan, M. *et al.* MamY is a membrane-bound protein that aligns magnetosomes and the motility axis of helical magnetotactic bacteria. *Nat. Microbiol.* **4,** 1978–1989, (2019).
13. Zwiener, T. *et al.* Identification and elimination of genomic regions irrelevant for magnetosome biosynthesis by large-scale deletion in *Magnetospirillum gryphiswaldense*. *BMC Microbiol.* **21,** 65, (2021).
14. Karimova, G., Pidoux, J., Ullmann, A. & Ladant, D. A bacterial two-hybrid system based on a reconstituted signal transduction pathway. *PNAS* **95,** 5752–5756, (1998).
15. Uebe, R. *et al.* The dual role of MamB in magnetosome membrane assembly and magnetite biomineralization. *Mol. Microbiol.* **107,** 542–557, (2018).

16. Kolinko, I. *et al.* Biosynthesis of magnetic nanostructures in a foreign organism by transfer of bacterial magnetosome gene clusters. *Nat. Nanotechnol.* **9,** 193–197, (2014).

17. Pfeiffer, D. *et al.* A bacterial cytolinker couples positioning of magnetic organelles to cell shape control. *PNAS* **117,** 32086–32097, (2020).

18. Raschdorf, O., Plitzko, J. M., Schüler, D. & Müller, F. D. A tailored *galK* counterselection system for efficient markerless gene deletion and chromosomal tagging in *Magnetospirillum gryphiswaldense*. *Appl. Environ. Microbiol.* **80,** 4323–4330, (2014).
