# Supplementary material for: MamF-like proteins are distant Tic20 homologs involved in organelle assembly in bacteria
Source: Nat Commun. 2024 Dec 9;15:10657. doi: 10.1038/s41467-024-55121-0 (PMC11628618; doi:10.1038/s41467-024-55121-0)
Supplement: Supplementary file 2 — Description of Additional Supplementary Files [file 41467_2024_55121_MOESM2_ESM.pdf]

Description of Additional Supplementary Files:

Supplementary Data 1: Accession numbers of proteins used for phylogenetic analyses of MFPs (Supplementary Fig. 1a) and the Tic20/HOTT superfamily (Supplementary Fig.1d).

Supplementary Data 2: Accession numbers of proteins used for CLANS analysis (Fig. 1b) and multiple sequence alignments (Fig. 1d).

Supplementary Data 3: Raw data for magnetosome crystal size (Fig.2a), magnetosome number per cell (Fig. 2b), qMNA (Figure 2d, e), and magnetotaxis analyses (Fig. 2f).

Supplementary Data 4: Proteins enriched or depleted in the ΔF3 MM fraction as determined by quantitative proteomic analysis of Fig. 3d and Supplementary Fig. 5b.

Supplementary Data 5: Raw data for magnetosome crystal size analysis for Fig. 5b.
