# Supplementary material for: MamF-like proteins are distant Tic20 homologs involved in organelle assembly in bacteria
Source: Nat Commun. 2024 Dec 9;15:10657. doi: 10.1038/s41467-024-55121-0 (PMC11628618; doi:10.1038/s41467-024-55121-0)
Supplement: Supplementary file 8 — Reporting Summary [file 41467_2024_55121_MOESM8_ESM.pdf]

nature portfolio

Corresponding author(s): René Uebe

Last updated by author(s): Nov 20, 2024

Reporting Summary

Nature Portfolio wishes to improve the reproducibility of the work that we publish. This form provides structure for consistency and transparency in reporting. For further information on Nature Portfolio policies, see our Editorial Policies and the Editorial Policy Checklist.

Statistics

For all statistical analyses, confirm that the following items are present in the figure legend, table legend, main text, or Methods section.

n/a	Confirmed	
☐	☒	The exact sample size (*n*) for each experimental group/condition, given as a discrete number and unit of measurement
☐	☒	A statement on whether measurements were taken from distinct samples or whether the same sample was measured repeatedly
☐	☒	The statistical test(s) used AND whether they are one- or two-sided<br>*Only common tests should be described solely by name; describe more complex techniques in the Methods section.*
☒	☐	A description of all covariates tested
☐	☒	A description of any assumptions or corrections, such as tests of normality and adjustment for multiple comparisons
☐	☒	A full description of the statistical parameters including central tendency (e.g. means) or other basic estimates (e.g. regression coefficient) AND variation (e.g. standard deviation) or associated estimates of uncertainty (e.g. confidence intervals)
☐	☒	For null hypothesis testing, the test statistic (e.g. *F*, *t*, *r*) with confidence intervals, effect sizes, degrees of freedom and *P* value noted<br>*Give P values as exact values whenever suitable.*
☒	☐	For Bayesian analysis, information on the choice of priors and Markov chain Monte Carlo settings
☒	☐	For hierarchical and complex designs, identification of the appropriate level for tests and full reporting of outcomes
☒	☐	Estimates of effect sizes (e.g. Cohen's *d*, Pearson's *r*), indicating how they were calculated

Our web collection on statistics for biologists contains articles on many of the points above.

Software and code

Policy information about availability of computer code

Data collection	Eclipse Ti2-E N-SIM E fluorescence microscope (Nikon) with NIS-Elements 5.01 software (Nikon, https://www.nikoninstruments.com/Products/Software), Zeiss EM 902A and Jeol JEM-1400 Plus electron microscopes with DigitalMicrograph software(Gatan https://www.gatan.com);
Data analysis	For data analysis we used the following programs and tools: HHpred Version: 57c8707149031cc9f8edceba362c71a3762bdbf8 (Zimmermann et al., 2018, http://toolkit.tuebingen.mpg.de/hhpred); Fiji 1.54f (Schindelin et al., 2012, http://fiji.sc); GraphPad Prism 7 (http://www.graphpad.com/, RRID:SCR_002798); Adobe Illustrator CS6 (Adobe Systems, http://www.adobe.com/products/illustrator.html); Fit-o-mat 0.752 (Möglich, 2018; https://www.moeglich.uni-bayreuth.de/en/fit-o-mat/); Pfam 37.0 (Mistry et al., 2021, http://pfam.xfam.org/), CLANS v29.05.2012 (Frickey and Lupas, 2004, https://www.eb.tuebitueb.mpg.de/protein-evolution/software/ clans/); MaxQuant 1.6.3.4. (Cox and Mann, 2008, http://www.biochem.mpg.de/5111795/maxquant); Perseus 1.6.2.2 (Tyanova et al., 2016, http://www.perseus-framework.org); SignalP 5.0 (Armenteros et al., 2019, http://www.cbs.dtu.dk/services/SignalP/); ΔG prediction server v1.0 (Hessa et al., 2007, http://dgpred.cbr.su.se/); TOPCONS 2.0 (Tsirigos et al., 2015, https://topcons.cbr.su.se/); MAFFT 7.474 (Katoh et al., 2019, http://mafft.cbrc.jp/alignment/server/); TrimAl 1.3 (Capella-Gutiérrez et al., 2009, http://trimal.cgenomics.org); IQ-Tree 1.6.11 (Trifinopoulos et al., 2016, http://www.iqtree.org/); iTOL 6.9.1 (Letunic and Bork, 2019, https://itol.embl.de/); Geneious 8.1.4 (Biomatters, https://www. geneious.com/); Image LabTM Software 6.0.1 (Bio-Rad http://www.bio-rad.com/en-us/sku/1709690-image-lab-software); DigitalMicrograph (Gatan https://www.gatan.com); NIS-Elements 5.01 (Nikon, https://www.nikoninstruments.com/Products/Software); GRAVY calculator v27.12.2011 (Kyte and Doolittle, 1982; http://www.gravy-calculator.de/)

For manuscripts utilizing custom algorithms or software that are central to the research but not yet described in published literature, software must be made available to editors and reviewers. We strongly encourage code deposition in a community repository (e.g. GitHub). See the Nature Portfolio guidelines for submitting code & software for further information.

Data

Policy information about availability of data

All manuscripts must include a data availability statement. This statement should provide the following information, where applicable:

- Accession codes, unique identifiers, or web links for publicly available datasets
- A description of any restrictions on data availability
- For clinical datasets or third party data, please ensure that the statement adheres to our policy

All data are avilable within the article and supplementary files. Source data are provided with this paper. Mass spectrometry proteomics data have been deposited to the ProteomeXchange server with the dataset identifier PXD032959.

Research involving human participants, their data, or biological material

Policy information about studies with human participants or human data. See also policy information about sex, gender (identity/presentation), and sexual orientation and race, ethnicity and racism.

Reporting on sex and gender	*Use the terms sex (biological attribute) and gender (shaped by social and cultural circumstances) carefully in order to avoid confusing both terms. Indicate if findings apply to only one sex or gender; describe whether sex and gender were considered in study design; whether sex and/or gender was determined based on self-reporting or assigned and methods used. Provide in the source data disaggregated sex and gender data, where this information has been collected, and if consent has been obtained for sharing of individual-level data; provide overall numbers in this Reporting Summary. Please state if this information has not been collected. Report sex- and gender-based analyses where performed, justify reasons for lack of sex- and gender-based analysis.*
Reporting on race, ethnicity, or other socially relevant groupings	*Please specify the socially constructed or socially relevant categorization variable(s) used in your manuscript and explain why they were used. Please note that such variables should not be used as proxies for other socially constructed/relevant variables (for example, race or ethnicity should not be used as a proxy for socioeconomic status). Provide clear definitions of the relevant terms used, how they were provided (by the participants/respondents, the researchers, or third parties), and the method(s) used to classify people into the different categories (e.g. self-report, census or administrative data, social media data, etc.) Please provide details about how you controlled for confounding variables in your analyses.*
Population characteristics	*Describe the covariate-relevant population characteristics of the human research participants (e.g. age, genotypic information, past and current diagnosis and treatment categories). If you filled out the behavioural & social sciences study design questions and have nothing to add here, write "See above."*
Recruitment	*Describe how participants were recruited. Outline any potential self-selection bias or other biases that may be present and how these are likely to impact results.*
Ethics oversight	*Identify the organization(s) that approved the study protocol.*

Note that full information on the approval of the study protocol must also be provided in the manuscript.

Field-specific reporting

Please select the one below that is the best fit for your research. If you are not sure, read the appropriate sections before making your selection.

[x] Life sciences [ ] Behavioural & social sciences [ ] Ecological, evolutionary & environmental sciences

For a reference copy of the document with all sections, see nature.com/documents/nr-reporting-summary-flat.pdf

Life sciences study design

All studies must disclose on these points even when the disclosure is negative.

Sample size	No statistical methods were used to predetermine sample size. We used numbers that are standard practice in the field by including at least three biological replicates. At least 100 cells were checked for each protein localization analysis with SIM microscopy. The i number of replicates and sample sizes is provided in methods, figure (legends), or supplementary information.
Data exclusions	No data were excluded from the analyses.
Replication	All TEM and fluorescent micrographs are representative of the strain grown under the stated conditions. Each Cmag measurement experiment included three independent cultures. The cellular fractionation experiments were repeated two or more times with similar results.
Randomization	Consistent with practices in the field, allocation of samples into experimental groups was not random. For experiments involving deletions of the MFP and other MAI genes, strains were generated from the indicated parental strain and examined in parallel in given growth conditions. Similarly, complementation experiments with wild-type, mutant, or gene fusions were conducted under similar conditions. Replicates were used to ensure consistency in the results. All magnetite crystals that were capable of being measured were counted and

	measured in randomly selected cells.
Blinding	Consistent with practices in the field, investigators were not blinded. In all cases, blinding is not feasible since the effect of culture condition or mutation is apparent during data collection. Additionally, all experiments were replicated and were well-controlled through inclusion wildtype,<br>mutant, and complemented strains. For comparison between WT and deletion strains, experiments were not examined in particular order.

Reporting for specific materials, systems and methods

We require information from authors about some types of materials, experimental systems and methods used in many studies. Here, indicate whether each material, system or method listed is relevant to your study. If you are not sure if a list item applies to your research, read the appropriate section before selecting a response.

Materials & experimental systems

n/a	Involved in the study
☐	☒ Antibodies
☒	☐ Eukaryotic cell lines
☒	☐ Palaeontology and archaeology
☒	☐ Animals and other organisms
☒	☐ Clinical data
☒	☐ Dual use research of concern
☒	☐ Plants

Methods

n/a	Involved in the study
☒	☐ ChIP-seq
☒	☐ Flow cytometry
☒	☐ MRI-based neuroimaging

Antibodies

Antibodies used	Primary antibodies used for Western immunoblot analyses were: Rabbit polyclonal anti-MamJ (Pineda Antibody Service, (Scheffel and Schüler, 2007)); Rabbit polyclonal anti-MamK (Pineda Antibody Service, (Katzmann et al., 2010)); Rabbit polyclonal anti-MamM (Pineda Antibody Service, (Uebe et al., 2011)); Rabbit polyclonal MamY (ProteoGenix, (Dziuba et al., 2020) Lot n° P190109-SL701464); Rabbit polyclonal anti-MamA (Taoka et al., 2006); Rabbit polyclonal anti-MamC (Pineda Antibody Service, (Schübbe et al., 2006)); Mouse monoclonal anti-GFP (B-2) (Santa Cruz Biotechnology, Cat# sc-9996; RRID: AB_627695). As Secondary antibodies we used Goat anti-mouse-HRP (Invitrogen, Cat# 32430 RRID: AB_1185566); Goat anti-rabbit-AP (Sigma-Aldrich, Cat# A3687; RRID: AB_258103).
Validation	The GFP antobody is a commercial that has been tested and validated by the manufacturer. Recognition of GFP-labeled magnetosome proteins by the anti-GFP antibody was tested by comparing WT or mutant strains that contain GFP expression constructs transferred through conjugation and inserted into the chromosome by mini transposons (Tn5 or Tn7) and untransformed parental strains on Western blots; a corresponding image is shown in Source Data Fig. 4e in the blue boxed area. Recognition of magnetosome proteins by the anti-MamJ, MamC, MamM, MamA, MamY antibodies was validated by comparing WT MSR-1 or MFP mutant cells with the M05 mutant strain that lacks all magnetosome gene operons on western blots. An exemplary image is shown in Source data Fig. 3a (M05 corresponds to the 4th lane after the leftmost marker).

Plants

Seed stocks	*Report on the source of all seed stocks or other plant material used. If applicable, state the seed stock centre and catalogue number. If plant specimens were collected from the field, describe the collection location, date and sampling procedures.*
Novel plant genotypes	*Describe the methods by which all novel plant genotypes were produced. This includes those generated by transgenic approaches, gene editing, chemical/radiation-based mutagenesis and hybridization. For transgenic lines, describe the transformation method, the number of independent lines analyzed and the generation upon which experiments were performed. For gene-edited lines, describe the editor used, the endogenous sequence targeted for editing, the targeting guide RNA sequence (if applicable) and how the editor was applied.*
Authentication	*Describe any authentication procedures for each seed stock used or novel genotype generated. Describe any experiments used to assess the effect of a mutation and, where applicable, how potential secondary effects (e.g. second site T-DNA insertions, mosiacism, off-target gene editing) were examined.*
